# The Role of Inhibitory Control in Moderating Working Memory Training Transfer: Evidence from a Preregistered Trial with Older Adults

**DOI:** 10.21203/rs.3.rs-9156758/v1

**Published:** 2026-06-22

**Authors:** Anja Pahor, Morgan Gomez, Susanne Jaeggi, Aaron Seitz

**Affiliations:** University of Maribor; Northeastern University; Northeastern University; Northeastern University

## Abstract

Most contemporary cognitive training programs incorporate gamified elements, yet the cognitive mechanisms through which gamification influences training and transfer remain poorly understood. Although gamification may enhance engagement, it can also introduce salient, task-irrelevant features that increase demands on executive control, particularly inhibitory control (IC). We therefore tested whether baseline IC moderates transfer following working memory training, hypothesizing that lower IC would constrain transfer by impairing the filtering of irrelevant information during training. We first examined this hypothesis in a secondary analysis of a published working memory training dataset in younger adults. We then tested its robustness in older adulthood in a preregistered, double-blind randomized crossover trial in which healthy older adults completed 40 sessions of N-back training in game and nongame conditions. The results of the latter showed that baseline IC significantly moderated transfer to Untrained N-back and Complex Span outcomes, whereas general cognitive ability did not. These findings identify IC as a key individual-difference factor in cognitive plasticity and suggest that baseline profiling may help tailor interventions for populations at risk of age-related cognitive decline.

## Introduction

Age-related cognitive decline manifests in several ways, such as reduced cognitive processing speed^1^, diminished inhibitory control^2^, and decreased working memory capacity^2^. These impairments can adversely affect multitasking, problem-solving^3^, and complex decision-making abilities, ultimately having a substantial impact on an individual’s everyday activities and quality of life as they age^4^. At the same time, there is notable variability among older adults, with some developing cognitive issues and others retaining their mental sharpness even in advanced age^5,6^.

Working memory (WM) training interventions have gained in popularity as tools to support cognition in aging^7–9^. WM training interventions in older adults have been shown to be successful in improving WM functions^10^ and leading to broader cognitive benefits and promoting neuroplasticity^11–13^. However, the literature shows mixed results, with some studies reporting significant transfer to untrained tasks following WM training in older adults^12,14–16^, while others indicate limited effects^10,13,17,18^, with meta-analyses generally demonstrating small effects overall for older adults^9,19^. Methodological differences, such as training duration^20^, training task selection^21,22^, and individual difference factors^23,24^ contribute to these inconsistencies. Training-induced gains also vary as a function of individual differences in factors such as age, motivation, personality, and genetic predisposition^25^.

Baseline cognitive ability is a frequently investigated source of heterogeneity in cognitive training outcomes. For instance, one study reported that, among older adults, both age and initial WM performance predicted the magnitude of improvement following WM training^26^. Two theoretical accounts explain how baseline cognitive ability influences the benefits older adults gain from cognitive training: the Compensation Account and the Magnification Account^27,28^. The Compensation Account suggests that individuals with lower baseline cognitive abilities have greater room for improvement and thus benefit the most from cognitive training. This perspective assumes that cognitive training compensates for deficits, enabling older adults with lower initial performance to achieve significant gains, which has been supported by a number of studies^29–32^. In contrast, the Magnification Account posits that older adults with higher baseline cognitive abilities are better equipped to utilize cognitive resources more efficiently and therefore gain the most from cognitive training, which has also received empirical support^33–35^. There is also evidence from WM training research indicating that the observed effects depend on the outcome measure. In a sample of older adults, magnification effects were reported for measures that require active information processing, whereas compensation effects were reported for measures that require a passive strategy^36^. Inconsistent support for these accounts suggests that the effectiveness of cognitive training depends on the interplay between an individual’s initial abilities, the nature of the intervention, and how outcomes are measured.

Another factor that may account for the limited and variable benefits of cognitive training is participants’ lack of engagement. Gamification has emerged as a promising strategy for enhancing motivation and engagement in learning contexts^37–39^ thereby potentially improving the efficacy of training programs. However, gamification of cognitive training has also yielded mixed results, with evidence suggesting that it may occasionally impair learning by introducing distractions^40,41^. Given that executive functions are modifiable through targeted intervention, systematically comparing Game versus Nongame cognitive training is critical for clarifying their differential effects on core cognitive systems and for optimizing intervention design. In particular, it is important to determine whether gamification primarily enhances motivational engagement, as suggested by prior research^39^, or whether such motivational gains also translate into measurable transfer outcomes. Furthermore, individual differences in responsiveness to gamification^42^ remain underexplored.

While several studies indicate that baseline cognitive abilities moderate transfer effects^25,43,44^, it is not known whether the effect of gamification on transfer varies as a function of individuals’ inhibitory control. We address this question and hypothesize that baseline inhibitory control will account for variability in transfer because successful performance in a game-based training context requires suppressing distractors and other task-irrelevant features to maintain focus on the core cognitive demands of training. Accordingly, individuals with lower baseline inhibitory control may benefit less from game training, showing weaker transfer relative to nongame training.

To provide an initial test of this hypothesis, we conducted secondary analyses of a published randomized controlled trial^45^ in which young adults were randomly assigned to Game or Nongame working memory training (Study 1). Consistent with our predictions, Study 1 yielded preliminary evidence that baseline inhibitory control performance moderates near transfer as a function of gamification, supporting the plausibility of inhibitory control as a mechanism underlying individual differences in responsiveness to Game training.

These questions may be particularly consequential in older adulthood, where game features may introduce added complexity and potential distractions that could overwhelm limited cognitive resources^46^ and reduce training benefits. We therefore expected moderation effects to be more pronounced in older adults, given both age related declines and substantial interindividual variability in inhibitory control^47^. To test this hypothesis, we conducted a double blind, preregistered randomized crossover clinical trial in which healthy older adults completed forty sessions of WM training using the N-back task, half of which were gamified (Study 2; Fig. 5). The N-back task is one of the most common tasks used in working memory training studies^48,49^. This task requires participants to continuously monitor a sequence of items and indicate when the current item matches the one presented 'N' steps back in the sequence, which requires constant updating of items in working memory. The first phase of the crossover matched the duration of typical working memory training interventions, including Study 1^45^, and therefore provided an opportunity to examine whether the effect of training condition Game vs Nongame) on transfer to untrained working memory tasks varies as a function of baseline inhibitory control. To evaluate the specificity of this moderation effect, we also tested general cognitive ability as a comparator moderator. We hypothesized that participants with lower inhibitory control would show stronger transfer following Nongame training, whereas participants with higher inhibitory control would show stronger learning and transfer to untrained working memory measures following Game training during the first phase of the crossover. Although estimating the overall crossover effect was not a primary aim, given the absence of a control group for this comparison, we hypothesized statistically significant improvements across all four primary outcome measures at post-test that would be maintained at follow-up.

## Results

### Study 1

**Fig. 1:**
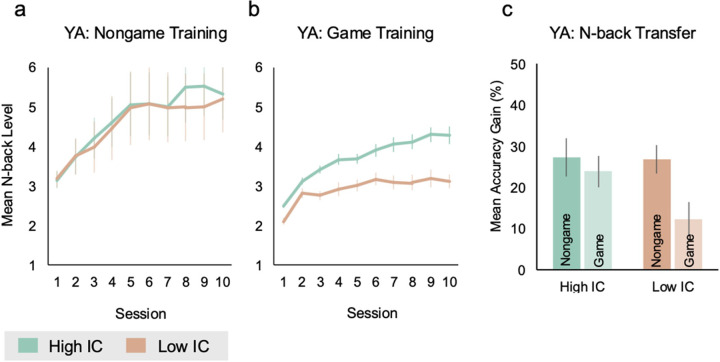
Training and Untrained N-back performance in a Young Adult (YA) population (Study 1). Each training session consisted of two 20-minute subsessions (total training time = 400 minutes), ***a*** Mean N-back training level achieved across 10 Nongame training sessions (N_HighlC_ = 50, N_LowlC_ = 75). ***b*** Mean N-back training level achieved across 10 Game training sessions (N_HighlC_ = 51, N_LowlC_ = 25). ***c*** Mean Untrained N-back accuracy gain as a function of group and training condition (High IC: N_nongame_ = 50; N_game_ = 50; Low IC: N_nongame_ = 75; N_game_ = 25). Error bars represent SEM.

Data from a published RCT with undergraduate students^45^ were re-analyzed to examine whether there is a difference in training performance and near transfer to a working memory task in groups assigned to Game and Nongame N-back training as a function of baseline performance on an inhibitory control (IC) task. For the Nongame training condition, there was no evidence of a statistically meaningful difference in Training Gain between individuals with low versus high baseline IC performance (BF_10_ = 0.20). In contrast, for the Game training condition, there was moderate evidence that individuals with higher baseline IC performance exhibited greater Training Gain than those with lower baseline IC (BF_10_ = 3.81; Fig. 1a–b).

We next examined near-transfer effects. Among individuals with high baseline IC, there was no evidence that transfer to an Untrained N-back task differed between the Game and Nongame training conditions (BF_10_ = 0.26). By contrast, within the low IC group, there was anecdotal evidence indicating greater transfer to the Untrained N-back task among participants assigned to the Nongame training condition compared with those in the Game condition (BF_10_ = 2.25; Fig. 1c). These findings suggest that gamification may be less effective in promoting near transfer among individuals with lower inhibitory control performance, even within a younger adult population. However, as inhibitory control was measured using only a single task and there was no measure of general cognitive ability, the groups were not evenly balanced, and the observed effect was anecdotal rather than a robust outcome, further investigation was needed. Given prior evidence that inhibitory control declines with age^2^, we hypothesized that this interaction would be more pronounced in older adults. To test this hypothesis, we conducted a more extensive preregistered clinical trial with an older adult population (Study 2).

## Study 2

### Training

#### Training - Before Crossover.

The first part of the pre-registered crossover study involving older adults (N = 73) was used to examine whether individuals with different levels of baseline IC show different training gains as a function of Training Condition (Game/Nongame). It should be noted that no control group was included, as the primary objective was to examine whether baseline IC affects study outcomes in older adults. Bayesian analyses provided no evidence for a meaningful difference in training gain between participants with low versus high baseline IC in either the Game condition (BF_10_ = 0.34) or the Nongame condition (BF_10_ = 0.66). In contrast to the pattern observed in the young adult cohort (Study 1), baseline IC did not appear to moderate training-related improvement in the older adult sample.

#### Training - Both parts.

**Fig. 2:**
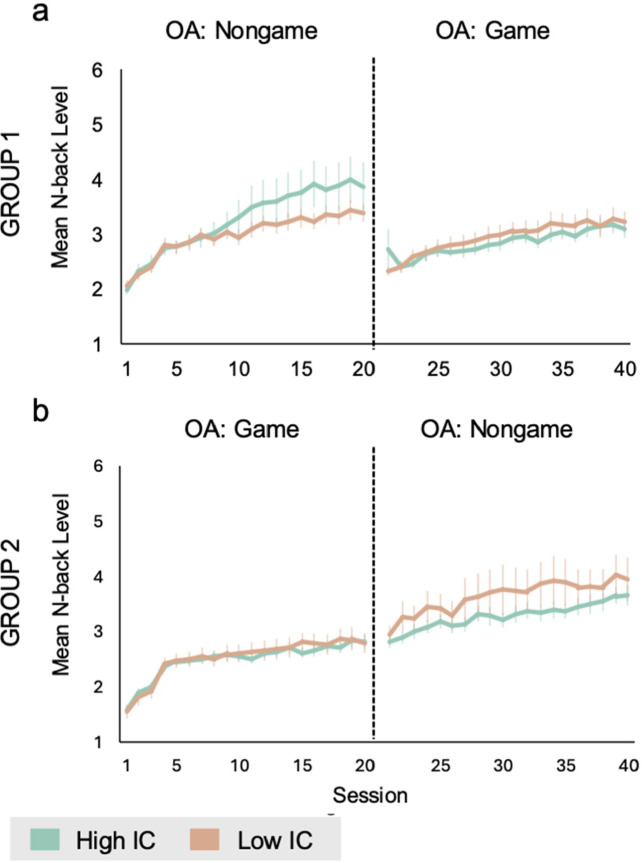
Training performance in an Older Adult (OA) population (Study 2; N = 73). Average N-back training performance across forty 20-minute long training sessions (total training time = 800 minutes), **a** Training performance for the Nongame condition and subsequent Game N-back training after crossover (N = 37), stratified by low and high baseline inhibitory control (IC). **b** Training performance for the Game condition and subsequent Nongame N-back training after crossover (N = 36), stratified by low and high baseline (IC). Error bars represent SEM.

As an exploratory aim, we examined whether training order influenced gains, comparing participants who completed Nongame training followed by Game training with those who completed Game training followed by Nongame training (Fig. 2). A Bayesian mixed repeated-measures ANOVA (Time: first vs. last training session; Training Condition: Nongame-first vs. Game-first) provided overwhelming evidence for a main effect of Time (BF^incl^ > 1000), indicating robust improvement in N-back training performance across forty sessions. Crucially, there was extremely strong evidence for a Time × Training Condition interaction (BF^incl^ = 16,136.93), suggesting that performance improvements differed markedly between groups. In Session 1, participants in the Nongame-first sequence showed better N-back training performance (*M* = 2.03, *SD* = 0.40) than those in the Game-first sequence (*M* = 1.56, *SD* = 0.44). By Session 40, this pattern had flipped: the Game-first sequence showed better N-back training performance (*M* = 3.78, *SD* = 1.21) than the Nongame-first group (*M* = 3.16, *SD* = 0.80). Thus, both groups improved over the course of the study, but the increase was larger for the Game-first group than for the Nongame-first group. Age was a meaningful predictor of training performance (BF^incl^ = 38.18), with older participants showing lower overall scores (posterior mean = − 0.031), whereas gender, depressive symptoms (GDS), and baseline inhibitory control did not improve model fit (all BF^incl^ < 1).

## Transfer: Primary Outcome Measures stratified by IC

**Fig. 3:**
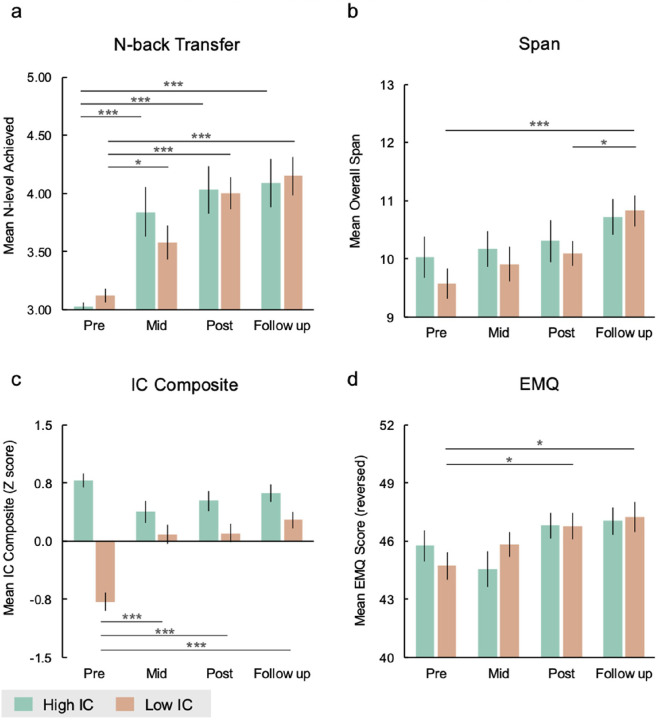
Mean performance on Study 2 primary outcome measures at four time points of measurement, split by IC performance at pre-test. Note that the cross-over took place after the mid-test, **a** Group mean of the maximum N-back level achieved on the Untrained N-back task across four assessment time points (N_HighlC_ = 35; N_LowlC_ = 35). **b** Mean Overall Span at each timepoint (N_HighlC_ = 34; N_LowlC_ = 34), calculated as the sum of the two correctly recalled highest sequence lengths in Simple and Complex Span tasks, **c** Mean reversed IC Composite (N_HighlC_ = 35; N_LowlC_ = 35), calculated as mean performance on Flanker and Rule switch tasks, with higher scores showing better performance, **d** Reversed EMQ score (N_HighlC_ = 32; N_LowlC_ = 32); higher score indicates less memory difficulties. Error bars represent SEM. * BF_10_: 3.0–9.9; ** BF_10_: 10.0–99.9; *** BF_10_ ≥ 100. IC: Inhibitory Control. EMQ: Everyday Memory Questionnaire.

As predicted, visual inspection of the data indicated distinct patterns of change across the four assessment time points (pre-test, mid-test, post-test, and follow-up) between the low and high inhibitory control (IC) groups (Fig. 3; **Supplementary Table 2)**. Bayesian repeated-measures ANOVAs were conducted separately for each group, followed by post hoc tests with posterior odds corrected for multiple testing. Because all four time points were included in the analyses, gamification was not entered as a factor given the crossover design. Moderation analyses reported further below complement these results by adopting a more continuous approach to baseline IC.

### Untrained N-back.

One participant was removed from the sample (|z| > 3). Highest N-level achieved ranged from 3 to 4 at pre-test and from 3 to 7 in subsequent testing sessions. *In the high IC group*, the Bayesian repeated-measures ANOVA provided very strong evidence for improvement in N-back performance as a function across assessment time points (BF^incl^ > 100). This pattern was corroborated by post hoc comparisons corrected for multiple testing, which indicated decisive evidence for improvement between pre-test and mid-test, pre-test and post-test, and pre-test and follow-up (BF^10,U^ for all three comparisons > 100; posterior odds > 100). Performance appeared to stabilize between post-test and follow-up, with only anecdotal evidence supporting further change (BF^10,U^ = 0.76, posterior odds: 0.31). *In the low IC group*, there was also very strong evidence for improvement in N-back performance across assessment time points (BF^incl^ > 100). Post hoc tests between pre-test and subsequent assessment timepoints showed strong evidence for improvement at mid-test (BF^10,U^ = 21.83; posterior odds = 9.04) post-test (BF^10,U^ > 100; posterior odds > 100) and follow-up (BF^10,U^ > 100; posterior odds > 100). There was no evidence of change in performance at follow-up relative to post-test (BF^10,U^ = 0.52, posterior odds: 0.21), suggesting maintenance of gains after the study had ended.

### Working Memory Span.

Four participants were excluded from analysis, one with missing Complex Span pre-test data, and 3 due to noncompliance (memory span ≤ 2 on simple or complex tests); there were no z-score outliers. Overall Span (sum of Simple and Complex Spans) ranged from 7 to 15 at pre-test and from 7 to 16 in subsequent testing sessions (N = 68). *In the high IC group*, a Bayesian repeated-measures ANOVA on Overall Span across pre-test, mid-test, post-test, and follow-up indicated anecdotal evidence for improvement over time (BF^incl^ = 1.85) therefore post hoc tests were not conducted. *In the low IC group*, the Bayesian ANOVA provided very strong evidence for improvement in Overall Span over time (BF^incl^ > 100). However, there was no evidence of improvement in Overall Span at mid-test (BF^10,U^ = 0.36; posterior odds: 0.15) and anecdotal evidence of improvement at post-test (BF^10,U^ = 3.08; posterior odds: 1.28). Results showed extreme evidence of improvement in Overall Span at follow-up compared to pre-test (BF^10,U^ > 100, posterior odds > 100). There was also strong evidence for improvement at follow-up relative to post-test (BF^10,U^ = 16.43, posterior odds: 6.08), indicating continued improvement in working memory performance after training had ended for unknown reasons (**Sub**).

### Inhibitory Control Composite.

Three participants was removed from the sample based on IC Composite performance (|z| > 3; N = 70). IC scores were reversed so that a higher value reflects better inhibitory control performance (Fig. 3c). *In the high IC group*, a Bayesian repeated-measures ANOVA on Overall Span across the four assessment timepoints showed no evidence of improvement in IC performance over time (BF^incl^ = 0.57). *In the low IC group*, the same analysis revealed very strong evidence in favor of improvement in IC performance over time (BF^incl^ > 100). Post hoc tests between pre-test performance and subsequent assessment points showed very strong evidence of improvement at mid-test (BF^10,U^ > 100; posterior odds: 148.90), post-test (BF^10,U^ > 100; posterior odds: 230.25), and follow-up (BF^10,U^ > 100; posterior odds: 73222.04). There was no evidence of a statistically significant difference between post-test and follow-up (BF^10,U^ = 0.34), suggesting maintenance of gains over time.

### Everyday Memory Questionnaire (EMQ).

Sixty-nine participants completed the EMQ survey at all four time points and 5 participants were excluded due to outliers (|z| > 3), resulting in a final sample of 64 participants. EMQ scores were reverse-coded so that a higher score indicates better self-reported memory performance, i.e. fewer memory difficulties (Fig. 3d). In the high inhibitory control group, a Bayesian repeated-measures ANOVA conducted across pre-test, mid-test, post-test, and follow-up indicated moderate evidence for an effect of Time (BF^incl^ = 6.09). However, post hoc comparisons involving pre-test did not yield evidence for change (all BF^10,U^ < 1). In the low inhibitory control group, the same analysis showed very strong evidence for an effect of Time (BF^incl^ = 88.85). Post hoc comparisons showed no evidence for change between pre-test and mid-test (BF^10,U^ = 0.96). In contrast, there was evidence for improvement from pre-test to post-test (BF^10,U^ = 7.37) and from pre-test to follow-up (BF^10,U^ = 10.17), which remained present after correction for multiple comparisons, as reflected in the posterior odds (3.05 and 4.21, respectively). No evidence of change in EMQ score was observed between post-test and follow-up (BF^10,U^ = 0.31).

## Transfer: Moderation

Moderation models were used to examine whether the effect of training condition (Game vs Nongame) on transfer to untrained working memory tasks differs across levels of baseline inhibitory control (IC) or general cognitive ability (GCA). IC and GCA were modeled as continuous moderators, allowing us to assess how training effects changed across the full range of individual differences. Untrained N-back, Simple and Complex Span were used as working memory outcome measures. In all models, we controlled for age, gender, depression, baseline performance on the dependent measure, as well as GCA or IC, respectively. Eight participants were excluded as outliers on IC/GCA tasks and noncompliance on Simple or Complex Span tasks (memory span ≤ 2) hence the analytical sample consisted of 65 participants.

### IC Model

#### N-back.

**Fig. 4:**
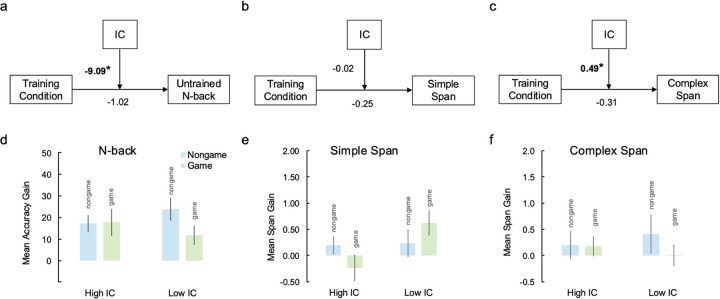
**a-c** A conceptual representation of the moderation models using unstandardized coefficients and IC as moderator in Study 2. Covariates include age, gender, GCA composite, depression score, and pre-test performance on the dependent measure, **d** Mean Untrained N-back accuracy gain at mid-test, divided into low and high IC groups with a median split, **e** Simple Span gain at mid-test, divided into low and high IC groups with a median split, **f** Complex Span gain at mid-test, divided into low and high IC groups with a median split. Error bars represent SEM. N = 65. IC: Inhibitory Control.

A moderation analysis was conducted to evaluate the influence of Training condition on untrained 2-back accuracy at midtest (hereafter referred to as Untrained N-back), with standardized inhibitory control (IC) included as a moderator (Fig. 4a). This approach aimed to assess the interactive effects of training condition and IC on Untrained N-back performance, while accounting for general cognitive ability and other factors. The overall model was significant (F(8,56) = 3.55, *p* < .01) with an R^2^ value of 0.34. The effect of Training condition on Untrained N-back at any value of IC was not statistically significant (*p* = 0.76). To test the hypothesis that IC moderates the effect of Training Condition on N-back transfer, we examined the interaction term, which was statistically significant (*b* = 9.09, *F*(1,56) = 8.10, *p* < 0.01). Simple slopes analyses showed that at low IC (− 1 SD), the Game condition showed lower N-back transfer than the Nongame condition (*b* = − 10.61, *p* = .03), whereas at high IC (+ 1 SD) the Game condition showed a trend toward higher N-back transfer (*b* = 8.35, *p* = .08). Johnson–Neyman results indicate that Game training yielded significantly lower Untrained N-back transfer than Nongame training at low IC (IC ≤ − 0.78 SD; ~lowest 20%), whereas it yielded significantly higher Untrained N-back transfer at very high IC (IC ≥ 1.25 SD; ~highest 7.7%), with no reliable condition differences across the intermediate IC range. Note that the moderation model treats IC as a continuous variable rather than the discrete median split used for visualization in Fig. 4d–f.

#### Simple Span.

The same moderation model was tested except with Simple span as the outcome variable (Fig. 4b). The overall model was significant (F(8,56) = 6.90, *p* < .01) with an R^2^ value of 0.50. There was no evidence that Simple Span transfer at mid-test depends on Training Condition (*p* = 0.23) at any level of IC, nor was there evidence of a statistically significant interaction between IC and Training Condition (*b* = −0.02, *F*(1,56) = 0.01, *p* = 0.94).

#### Complex Span.

The overall model was significant (*F*(8,56) = 6.40, *p* < .01) with an R^2^ value of 0.48 (Fig. 4c). There was no evidence that Complex Span transfer depends on Training Condition (*p* = 0.21) at any level of IC. To test the hypothesis that IC moderates the effect of Training Condition on Complex Span transfer, we examined the interaction term, which was statistically significant (*b* = 0.49, *F*(1,56) = 4.22, *p* = 0.04). Training Condition significantly predicted Complex Span transfer among participants with low inhibitory control (−1 SD, *b* = −0.83, *p* = 0.02), but not among those with high inhibitory control. Johnson-Neyman Analyses showed that the effect of Training condition was significant at IC values below − 0.46 SD, which represents 30.8% of the sample. These results suggest that individuals with low baseline IC showed more transfer to Complex Span when assigned to Nongame N-back training (Fig. 4c).

## GCA Model

To test the alternative hypothesis that GCA would be a better predictor of the outcomes of the intervention than IC skills, the same moderation models were run on N-back, Simple and Complex Span except that GCA was the moderator and IC the covariate, along with other covariates: age, gender, depression, and pre-test performance on the test of interest. For *N-back*, the overall model was significant (*F*(8,56) = 2.25, *p* = .04) with an R^2^ value of 0.24. There was no evidence that the effect of Training Condition on Untrained N-back performance (at any value of GCA) was statistically significant (*p* = −1.14), nor was there evidence of a statistically significant interaction between Training Condition and GCA (*b* = 3.37, *F*(1,56) = 0.15, *p* = 0.70). Likewise for *Simple Span*, the overall model was significant (*F*(8,56) = 7.36, *p* < 0.001) with an R^2^ value of 0.51, however there was no evidence that the effect of Training Condition on Simple Span performance (at any value of GCA) was statistically significant (*p* = 0.19), nor was there evidence of a statistically significant interaction between Training Condition and GCA (*b* = 0.65, *F*(1,56) = 1.86, *p* = 0.18). For *Complex Span*, while the overall model was significant (*F*(8,56) = 5.78, *p* < 0.001) with an R^2^ value of 0.45, there was no evidence that Training Condition affected Complex Span performance at any value of GCA (*p* = 0.19), nor was there evidence of a statistically significant interaction between Training Condition and GCA (*b* = 0.71, *F*(1,56) = 1.42, *p* = 0.24). In sum, there is no evidence that GCA moderates the relationship between Training Condition (gamification) and transfer to measures of working memory performance, after accounting for pre-existing IC ability and other relevant factors.

## Secondary and Other Outcomes

### Training Experience (TE).

Because differences in user experience or perceived difficulty could influence engagement and, in turn, performance on transfer tasks, we examined participants’ subjective evaluations split by IC and by Training Condition. Self-reported training evaluations were collected at the end of the final training session in Part 1 (i.e. session 20) and Part 2 (i.e. session 40) of the study. Bayesian independent-samples t-tests provided mostly evidence in favor of the null hypothesis for differences in Training Experience (TE) outcomes between groups **(Supplementary Table 3**). For the IC comparison, all Bayes factors at the end of Part 1 of training were below 1 (BF_10_ = 0.25–0.55), indicating no meaningful differences between High and Low IC groups on training enjoyment, difficulty, progress, or interface. For the gamification comparison, evidence favored the null hypothesis for most outcomes (BF_10_ = 0.26–0.49), with two exceptions. At the end of Part 1 of training (before the crossover), participants in the Nongame training condition reported a better user experience with the interface (“The interface worked the way I wanted, for example, I was able to select the items I wanted to”) than participants in the Game condition (BF_10_ = 5.41). By the end of Part 2 of training, when participants had completed both training conditions, there was anecdotal evidence for higher enjoyment of Game training (BF_10_ = 2.55) compared to Nongame training. All remaining comparisons indicated no reliable group differences.

When comparing low and high IC groups *within* each Training Condition, there was no evidence for group differences in training enjoyment, perceived difficulty, progress, or interface usability at the end of Part 1 (Nongame-first: BF_10_ = 0.33–0.47; Game-first: BF_10_ = 0.36–0.67). Following the crossover to Part 2, no differences were observed for participants who switched to Game training (BF_10_ = 0.33–0.43). In contrast, among participants who switched to Nongame training, the low IC group reported greater perceived difficulty (BF_10_ = 2.12; High IC: *M* = 7.80, *SD* = 1.01; Low IC: *M* = 8.56, *SD* = 1.03) and slightly poorer interface usability than the high IC group (BF_10_ = 2.36; High IC: *M* = 4.70, *SD* = 0.47; Low IC: *M* = 4.06, *SD* = 1.12); however, the evidence supporting these effects was weak.

### Exit Interview.

The responses to questions regarding memory, concentration, and multitasking were coded and are presented **Supplementary Fig. 1b**. While most participants did not report noticeable changes in their daily lives, those who did primarily noted improvements in memory, such as better recall of names, phone numbers, passcodes, songs, and foreign language vocabulary. A quarter of participants also reported improvements in their ability to focus, for example being able to concentrate longer on readings, audiobooks, or paying more attention to driving. When asked about any other changes, positive or negative, that occurred since they began training, 53.4% of participants reported positive changes, 5.5% negative changes, 1.4% reported both, and 39.7% reported no change. Among the four individuals who reported negative experiences, one disliked computer games in general, two expressed frustration with memorizing long sequences, and one became more aware of his/her shortcomings in memory. When asked about which training game they preferred, 45.2% of participants stated that they preferred the game, 32.9% preferred the nongame, 4.1% stated they liked both and 17.8% gave no response or misunderstood the question.

## Discussion

This study systematically examined whether and how training condition (Game vs. Nongame) and individual differences, specifically inhibitory control (IC) and general cognitive ability (GCA), influence working memory training outcomes in younger and older adults. By characterizing factors associated with successful learning and transfer, and testing whether these effects differ across individuals, we aimed to generate evidence that can guide the design of training interventions that are better aligned with users’ cognitive profiles.

Results from Study 1 in a younger adult sample provided preliminary evidence that baseline inhibitory control performance moderates both training gains and near transfer, indicating that gamification may be less effective for individuals with lower inhibitory control even in early adulthood. The findings, based on an ad hoc analysis, were tentative due to limited measures and uneven group balance, so further research was needed.

The subsequent preregistered clinical trial in older adults (Study 2) showed that participants improved on the trained task; however, the trial did not replicate the training-related pattern observed in the younger adult sample. Specifically, there was no evidence that training gains differed as a function of baseline inhibitory control and gamification. The reason for this is unknown, although a possible explanation might be that older adults adapt to game challenges by focusing more deliberately, or adopting conservative strategies, which could in turn reduce the impact of baseline inhibitory control on training progress (but not necessarily on transfer).

Although estimating the overall crossover effect was not a primary aim given the absence of a control group for this comparison, we nevertheless preregistered and conducted analyses examining changes in four primary outcome measures from pre-test to post-test and follow-up to characterize overall transfer and maintenance effects. Across the four assessment time points, participants showed clear improvement on the Untrained N-back task, with mean achieved N level increasing substantially from pre to mid and continuing to rise through post and follow-up. Although the high inhibitory control group showed an early advantage in N-back transfer at mid assessment, the low inhibitory control group largely caught up by post and follow-up, suggesting that baseline inhibitory control was more strongly related to the rate of early learning than to final training performance. Transfer to the Overall Span measure was more modest and appeared to be driven primarily by the low inhibitory control group, emerging at follow-up rather than immediately post training. This delayed pattern may reflect consolidation processes or a slower translation of training gains to untrained working memory outcomes^50,51^. The inhibitory control composite showed the strongest time-related change in the low inhibitory control group, which improved markedly from pre to mid assessment and maintained higher scores at post-test and follow-up. In contrast, the high inhibitory control group remained relatively stable over time, suggesting greater scope for improvement among participants with initially lower inhibitory control, consistent with the Compensation account of cognitive training^27^. Finally, self-reported everyday memory functioning improved from pre- to post-test and follow-up in the low inhibitory control group, with little evidence of change in the high inhibitory control group. Taken together, these results align with prior work in showing the strongest effects on measures most proximal to the training task, with weaker and more variable effects on broader transfer outcomes^49^ such as inhibitory control and everyday functioning.

Nevertheless, at the follow-up session, 32% of older adults self-reported improvements in daily functioning such as language learning, painting, recalling medical results, and reading, suggesting that some participants experienced meaningful benefits beyond laboratory measures even when objective far transfer effects were modest.

To evaluate our primary preregistered predictions regarding who benefits most from Game training, we next tested a set of preregistered moderation models examining whether the effect of training condition (Game vs Nongame) on transfer to untrained working memory outcomes varies as a function of baseline inhibitory control or general cognitive ability. Across outcomes, the overall regression models explained a moderate to large proportion of variance after controlling for age, gender, depressive symptoms, baseline performance on the outcome measure, and general cognitive ability. However, evidence for differential effects of gamification was somewhat limited and depended on the outcome measure.

For the Untrained N back outcome, there was no overall main effect of training condition across the sample. Instead, the results were consistent with an interaction between inhibitory control and training condition. Specifically, participants with lower inhibitory control showed lower Untrained N back gains in the Game condition compared to the Nongame condition, whereas at the highest levels of inhibitory control the direction of the effect appeared to reverse, with Game training associated with greater transfer. This latter effect was observed only at very high inhibitory control values representing a small proportion of the sample, and differences between conditions were not reliable across the intermediate inhibitory control range. These findings therefore provide preliminary support for the possibility that gamification related transfer effects depend on baseline IC, although the pattern appears to be confined to the tails of the inhibitory control distribution.

For the span outcomes, evidence was mixed. There was no indication that the effect of gamification on Simple Span transfer differed across levels of IC. In contrast, for Complex Span we observed a significant inhibitory control by training condition interaction, such that participants with lower inhibitory control showed greater transfer in the Nongame condition. One possible explanation is that gamification introduces additional demands on attentional regulation, which may be more consequential for Complex Span tasks that require both storage and ongoing processing, rather than simple span tasks that primarily index short term storage capacity with minimal updating^52^. Consistent with this interpretation, transfer from N-back training to short term memory capacity tasks is often limited^21,34,53^.

When general cognitive ability was tested as an alternative moderator, there was no evidence that it moderated the relationship between training condition and transfer for any outcome. This suggests that the observed moderation effects are not readily explained by broad cognitive ability differences captured by the general cognitive ability composite.

In conclusion, this study suggests that baseline inhibitory control, rather than general cognitive ability, moderates the effects of gamification on transfer to working memory outcomes in older adults. These findings highlight the value of assessing baseline inhibitory control, especially reaction time switch costs, to tailor interventions to individual needs. More broadly, the results contribute to a more nuanced understanding of variability in cognitive plasticity in later life and motivate future trials that use baseline cognitive profiles to optimize intervention design, with the longer term goal of supporting cognitive health in populations at elevated risk for Alzheimer’s disease and related dementias.

## Methods

### Study 1

Data from a previously published study involving undergraduate students^45^ was analyzed to examine the extent to which baseline inhibitory control capacity interacts with gamification of the training paradigm. The sample included 217 participants (M = 19.95 years, SD = 2.36), of whom 59.9% identified as female, 38.7% as male, and 1.4% as unknown or other. Participants were randomly assigned to one of several training conditions in a 20-session N-back cognitive training study; the present analysis focuses specifically on the Game versus Nongame training conditions. Only participants who completed all 10 training sessions, each of which consisted of two 20-minute sessions, were included in the analysis (N^game^ = 76; N^nongame^ = 125). Near transfer was assessed using an Untrained N-back task (same task as in Study 2), while inhibitory control (IC) was indexed by reaction time on correct incongruent trials of a Countermanding task^54^. One outlier (|z| > 3) was removed based on performance on the Untrained N-back task.

### Study 2

#### Participants

A total of 116 individuals expressed interest in the study; 16 did not meet the screening criteria and were excluded, resulting in 100 enrolled participants. Of these, 27 withdrew or did not complete the study. The final clinical trial sample comprised 73 adults (83% Female, 84% White, M = 69 yrs, SD = 7.16). Individuals with self-report memory issues were allowed to participate, as long as they had not received any formal diagnosis of Alzheimer’s Disease and Alzheimer’s Disease–Related Dementias. The study was approved by the UC Riverside Institutional Review Board. Participants provided informed consent and received a digital $150 Amazon gift card for compensation.

#### Procedure

**Fig. 5:**
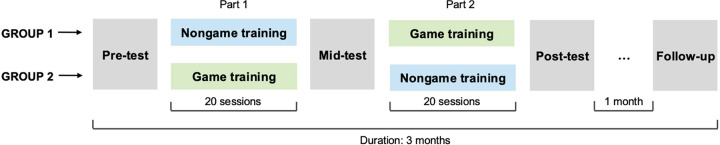
Study 2 Timeline.

Participants were screened over the telephone (for inclusion and exclusion criteria, see **Supplementary Table 1**). All procedures were conducted remotely: testing materials were mailed to participants, and sessions were completed either via videoconferencing or independently. Study 2 was preregistered as Arm 1 (https://clinicaltrials.gov/study/NCT05396586). The study consisted of 51 experimental sessions over three months, with training and testing conducted remotely on tablets using in-house software (BGC Science and Recollect the Study) and Qualtrics (Qualtrics, Provo, UT) for surveys and screening. Assessment tasks were administered on tablets at pre-test, mid-test, post-test, and a 1-month follow-up and were supervised on a videoconferencing platform by a researcher. The tablet-based working memory training intervention consisted of 40, 20-minute sessions in a cross-over design (Fig. 5). Participants were randomly assigned to two groups, one group trained on the Nongame task for 20 sessions and then after mid-test, switched to 20 sessions of Game training, whereas the other group completed the same tasks but in the opposite order (counterbalanced across participants). Participants were encouraged to play up to 2 sessions per day, with a goal of completing 10 per week. The first three training sessions in each training condition were remotely supervised via videoconferencing by a researcher. Once the participant met criteria to continue training without supervision, e.g. they demonstrated sufficient understanding of the training task and were able to level up at least once independently, they were able to continue training unsupervised.

#### Training

##### N-back.

In this task, participants memorize and update serial positions “n steps back” in a continuous stimulus stream. The Nongame condition is an N-back with 6 colors in which participants respond to targets appearing for 3.75 seconds with an ISI of 0.45 seconds by tapping the screen. The Game condition is a side-scroller platform game with the same mechanics as Nongame except that the participant controls a character that collects or avoids 6 objects with unique colors, shapes, and sounds based on whether they match the object presented n-trials previously. Both training approaches use the same general methods where each session consists of ten ~ 2-minute blocks of adaptive N-back training with task difficulty (i.e., n-level) determined by an adaptive procedure based on online session-based weighted accuracy and with user-paced breaks between blocks. Participants received continuous feedback on their training progress, along with summary feedback at the end of each run and a comprehensive summary at the conclusion of each session.

#### Primary Outcome Measures

##### Untrained N-back^55^.

A visual N-back task with two versions, featuring pictures of animals (A) or pictures of fruits and vegetables (B), the order of which was counterbalanced across participants (ABAB or BABA). All participants completed N-levels 1–3 and progression to further levels was contingent on performance on the previous level. The main outcome measures were change in 2-back accuracy and change in the highest N-level achieved at each timepoint compared to pre-test.

##### Working Memory Span.

Consisted of two adaptive tasks, Simple and Complex Span. Simple Span was used to assess visuospatial WM. A sequence of gophers appeared in 12 possible locations and the participants were asked to reproduce the sequences in the same order. In Complex Span, the same procedure was used, except that between each appearance of a gopher, participants completed a secondary sorting task. The outcome measure was change in Overall Span, calculated as the sum of the two highest set sizes that were recalled correctly in Simple and Complex Span tasks.

##### Inhibitory Control Composite.

Consisted of Flanker and Rule Switch, which were modeled after NIH Examiner Flanker^56^. Flanker consisted of 64 congruent and incongruent arrow trials (half of each, intermixed). The main outcome measure was the difference in median reaction time between correctly answered incongruent and congruent trials. In Rule Switch, participants matched a stimulus (red/blue square or circle) at the top of the screen to one of two stimuli in the lower corners based on either color or shape. The task included two single-rule blocks (20 trials each: shape and color) and a mixed block (64 trials) that alternates between rules. The primary measure was the difference in median reaction time between correct switch and non-switch trials. In UCancellation^54^, a tablet-based task that measures selective attention and inhibitory control, the outcome measure was concentration performance (ΣHits − ΣFalse alarms). This task was not included in the IC composite due to negative and nonsignificant correlations with the main outcome measures for Flanker (r = −0.07, p = 0.54, N = 70) and Rule Switch (r = −0.14, p = 0.24, N = 70) after removing outliers (|z|<3).

##### Everyday Memory Questionnaire Revised (EMQ).

The EMQ^57^ consists of 13 items on a 5-point likert scale that describe everyday events that might involve forgetting. The minimum score is 0 and the maximum is 52. To ensure consistency with other outcome measures in which higher scores indicate better cognitive performance, the EMQ scores were reverse-coded, such that higher values reflect fewer reported memory difficulties.

#### Secondary and Other Outcome Measures

A Training Experience Survey and an Exit Interview were preregistered as secondary outcome measures, while a battery of tests known as the Standard Older Adult Cognitive Battery (SOACB) was preregistered as an Other Pre-specified Measure to estimate general cognitive ability (GCA). The SOACB consists of six tablet-based tests, Word List Learning, Complex Figure, Picture Naming, Shipley-2 Vocabulary, Trail Making, and UCMRT, which were administered at pre-test and at follow-up.

##### Training Experience (TE).

The training experience survey was administered at the end of session 20 and at the end of session 40. It consists of 5 items that assess the participants’ perceptions and satisfaction with their cognitive training tasks. The survey consisted of 4 outcome measures: enjoyment, difficulty, subjective progress, and interface. Scores range from 1 to 5, with 1 being “not at all” and 5 being “very much”. Difficulty has a score range from 1 to 10, due to two items falling into this category, and a higher score indicates greater perceived difficulty.

##### Exit Interview.

After the follow-up assessment, participants were asked 5 open-ended questions about their subjective experience of participating in the study: (1) Since conducting the training, have you observed a change in your ability to concentrate? If so, please describe with examples, (2) Since conducting the training, have you observed a change in your ability to memorize information? If so, please describe with examples, (3) Since conducting the training, have you observed a change in your ability to multitask? If so, please describe with examples, (4) Are there any other changes, positive or negative, that you have noticed since you began training? If so, please describe with examples, and (5) Tell me about which training task you preferred, and why? (Tasks can be referred to by the astronaut or the circles).

##### Word List Learning.

Participants see a list of 10 words on the tablet, one at a time, and are instructed to read each word aloud as it appears and to remember them for later. Next, in the recognition phase, participants are shown 20 words one at a time and must indicate whether each word was part of the original list by tapping “yes” or “no” on the tablet. Finally, during the delayed recall phase, participants are asked to say as many words as they can, with their responses recorded by the tablet. Each participant is tested on three word lists. Main outcome measures are recognition and recall accuracy across three lists.

##### Complex Fig. 5 ^8,59^.

The Complex Figure Task assesses visuospatial abilities, visual memory, and organizational skills. Participants are shown an abstract figure on the left side of the screen and are asked to replicate it by drawing on the right side. After a delay, they are instructed to draw the figure again from memory. This process is repeated three times with different complex figures. The drawings were uploaded to a custom-made scoring portal and were evaluated by at least two independent raters, with an expert reviewer resolving discrepancies and providing a final score. The primary outcome measure is the final score for delayed recall, summed across all three trials.

##### Picture Naming^60^.

A vocabulary test in which participants are asked to name 23 common objects presented as black-and-white line drawings. The set consists of 15 items used in the CERAD version of the Boston Naming Test^61^ and 8 items used in the original and/or other revised versions of the test^62–64^ using in-house artwork. Images of the drawings are shown on the screen one by one, and participants must say the name of each image out loud with their responses recorded by the tablet. The audio recordings were evaluated by at least two independent raters, with an expert reviewer resolving discrepancies and providing a final score. This task measures language and word retrieval ability by scoring the responses to find the number of correctly named objects.

##### Shipley-2 Vocabulary Test^65^.

This measure of crystallized ability consists of 40 multiple-choice items and participants are asked to select the one word out of four choices that is closest in meaning to a target word. The main outcome measure is percentage of correctly solved problems.

##### Trail Making^66^.

Participants place their tablet on a flat surface in portrait orientation. In part A, they draw lines connecting numbers in order (1 to 2, 2 to 3, etc.). In part B, they alternate between numbers and letters (1 to A, A to 2, 2 to B, etc.) until the task is complete. This task measures cognitive functions such as visual attention, processing speed, mental flexibility, and executive function. The outcome measure used here is completion time of Part B (in seconds).

##### UCMRT^67^.

UCMRT Set D, which consists of 18 problems specifically designed to accommodate older adults and individuals with potential vision difficulties, was used to assess non-verbal abstract problem-solving ability. The outcome measure is the percentage of correctly solved problems.

#### Data analysis

Custom python scripts, IBM SPSS Version 29 (IBM corp.), and JASP 0.18.3 (JASP Team, 2019) were used for statistical analyses. Training Gain was calculated as the difference in mean N-back level achieved in the last 40-minutes of training compared to the first 40 minutes of training (for both studies). In Study 2, one participant was identified as an extreme outlier on Sessions 9–20; to retain this participant without undue influence, their scores were capped by replacing them with values corresponding to 3 standard deviations from the mean. The inhibitory control (IC) composite was calculated as the mean of Flanker congruency cost and Rule Switch switch cost where the unit of measurement is milliseconds. The general cognitive ability composite (GCA) was calculated as the mean of z-transformed scores for SOACB tests, namely Word List Recall, Complex Figure Recall, Picture Naming Test, Shipley Vocabulary Test, Trail Making Part B, and UCMRT at pre-test and follow-up. For the latter, z-scores were calculated based on pre-test means and standard deviations.

To examine transfer on the primary outcome measures across four time points (pre, mid, post, follow-up), Bayesian repeated-measures ANOVAs were conducted separately within the low- and high-IC groups, followed by post hoc comparisons with posterior odds adjusted for multiple testing. To test moderation, we estimated regression-based moderation models using the PROCESS macro for SPSS (Model 1), as described by Hayes (2018). These models evaluated whether the effect of training condition (Game vs. Nongame) on transfer to untrained working memory outcomes varied as a function of baseline IC or GCA. IC and GCA were modeled as continuous moderators to capture training effects across the full range of individual differences. Transfer outcomes comprised performance on an Untrained N-back task and on Simple and Complex Span measures, as these tasks engage working memory processes closely aligned with the trained domain. Other primary outcomes (e.g., inhibitory control or self-report data) were not included in the moderation analyses because the present models were specifically designed to test transfer within the working memory domain rather than broader or conceptually distinct constructs. All models included age, gender, depressive symptoms, and baseline performance on the corresponding outcome as covariates, as well as the alternate composite score (i.e., GCA in IC moderation models; IC in GCA moderation models).

## Supplementary Material

Supplementary Files

This is a list of supplementary files associated with this preprint. Click to download.

- SupplementaryInformation.pdf

## Data Availability

The datasets generated for this study are available at https://osf.io/5suzx/overview.
